# Calcium chloride induces dose-dependent architectural, mechanical, and transcriptional remodeling in *Pseudomonas putida* biofilms: an exploratory multi-scale study

**DOI:** 10.3389/fmicb.2026.1849493

**Published:** 2026-06-05

**Authors:** Zhibiao Yao, Yan Wang, Han Yu, Huiyu Liu, Keying Guo, Olivier Habimana

**Affiliations:** 1 Biotechnology and Food Engineering Program, Guangdong Technion-Israel Institute of Technology, Shantou, China; 2 Department of Chemistry, Southern University of Science and Technology, Shenzhen, China

**Keywords:** adhesins, atomic force microscopy, biofilm, calcium signaling, extracellular polymeric substances, *Pseudomonas putida*, species-specific adaptation, transcriptomics

## Abstract

Calcium ions (Ca^2+^) are known to enhance biofilm structural integrity in many bacteria, including *Pseudomonas aeruginosa*, by cross-linking exopolysaccharides. Still, their role in the soil bacterium *P. putida* remains largely unexplored. Here, we investigated the dose-dependent effects of calcium chloride on *P. putida* KT2442 biofilm architecture, matrix properties, nanomechanics, and global gene expression. Contrary to the stabilizing role observed in *P. aeruginosa*, calcium induced complex biphasic responses in *P. putida*. Calcium elicited complex, non-monotonic effects: while 1.5 mM CaCl₂ increased biofilm biovolume, a moderate 3 mM concentration limited architectural expansion and corresponded with the lowest measured biofilm stiffness in exploratory AFM assays. At 15 mM, biofilm thickness increased, but mechanical rigidity did not fully recover, and the molecular weight of matrix polysaccharides decreased. Transcriptomic analysis revealed dose-dependent reprogramming affecting approximately 3,600 genes at 3 mM and 15 mM calcium, with downregulation of genes involved in exopolysaccharide biosynthesis, large adhesins (*lapA*, *lapF*), and motility, alongside upregulation of stress response and ribosomal biogenesis pathways. These correlative findings suggest that calcium may act not merely as a structural ion but as a potent environmental signal that triggers species-specific adaptive responses correlated with altered biofilm architecture, mechanics, and transcriptional networks in *P. putida*. The direct causal links and the unique responses of calcium in comparison to other divalent cations have yet to be confirmed.

## Graphical abstract

**Figure:**
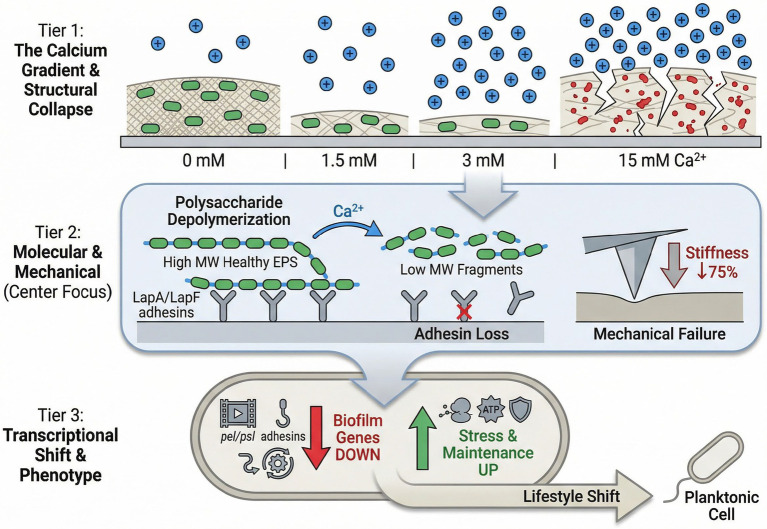
Ca^2+^: From structural ion to transcriptional signal in *P. putida*. Elevated calcium destabilizes biofilm architecture, weakness the matrix, and reprograms cells toward a planktonic, stress-resistant state.

## 1 Introduction

Bacteria commonly thrive not as isolated cells but as organized, surface-associated communities known as biofilms. These multicellular aggregates are encased within a self-produced, protective matrix of extracellular polymeric substances (EPS), which forms a complex and dynamic hydrogel (Boudarel et al., 2018; Flemming et al., 2007). Far from being a mere scaffold, this EPS matrix is a functionally active compartment that confers critical advantages: it provides mechanical stability, facilitates surface adhesion, acts as a diffusion barrier, and shelters resident cells from a range of environmental assaults, including antimicrobials, immune responses, desiccation, and osmotic fluctuations (Balducci et al., 2023; Flemming et al., 2007). The pervasiveness and durability of biofilms highlight their significant influence in various domains. They are central actors in chronic infections and industrial biofouling, presenting significant challenges, yet they also drive beneficial processes in environmental bioremediation, wastewater treatment, and sustainable bioprocessing (Flemming et al., 2016; Hobley et al., 2015).

Recent extensive research has significantly broadened and deepened our understanding of the sophisticated mechanisms governing species-specific biofilm regulation, revealing previously overlooked complexities. For instance, Wang et al. (2025) used ARTP mutagenesis to identify genes important for biofilm regulation in *Pseudomonas fluorescens*, demonstrating that even closely related pseudomonads can employ distinct genetic programs for matrix production. These findings demand focused studies on specific species to truly capture their unique ecological traits.

Multi-scale mechanical characterization methods, as recently discussed regarding extracellular matrices (Han et al., 2025), offer frameworks for understanding the hierarchical mechanical characteristics of hydrated biopolymer networks like bacterial biofilms. The structure of the biofilm matrix and its functional activity is largely regulated by the physico-chemical environment in which it develops, notably the availability of divalent cations. These ions, such as calcium (Ca^2+^), act as ionic bridges that cross-link negatively charged functional groups within the EPS network, primarily carboxylates on polysaccharide chains (Borchard et al., 2001). This phenomenon of cross-linking is acknowledged to enhance the interconnectivity of the matrix, increase structural integrity, and strengthen the comprehensive framework of the biofilm (Borchard et al., 2001). This paradigm has been extensively characterized in the opportunistic pathogen *Pseudomonas aeruginosa*, where exogenous calcium is known to strengthen alginate-based biofilms, contributing to their notorious persistence in clinical settings like the cystic fibrosis lung (Jacobs et al., 2022). Consequently, calcium has been broadly regarded in microbiology as a universal structural stabilizer of pre-formed bacterial biofilms.

However, generalizing this model across the bacterial domain may be an oversimplification. Bacterial adaptations to environmental stimuli are finely tuned to particular ecological contexts and life strategies. The versatile and robust soil bacterium *Pseudomonas putida* effectively challenges this paradigm. Unlike its pathogenic relative, *P. putida* is a root-colonizing beneficial microbe, playing vital roles in plant growth promotion, rhizosphere ecology, and the biodegradation of environmental pollutants (Molina et al., 2020). It inhabits diverse and fluctuating soil and aquatic environments where calcium availability can vary significantly. The role of calcium in the essential physiology of *P. putida* biofilm formation is notably under-researched, even though it holds significant ecological and biotechnological relevance. Preliminary evidence suggests that bacterial interpretations of ionic signals can be highly species-specific, pointing to a sophisticated interplay between ion sensing, transcriptional regulation, and phenotypic output that extends beyond simple physicochemical cross-linking (Parker et al., 2016).

This knowledge gap raises a pivotal question: In *P. putida*, do calcium ions function solely as a passive structural component of the biofilm matrix, or do they also act as a potent environmental signal that triggers a broader genetic reprogramming, ultimately steering the biofilm lifecycle? The specific mechanisms by which calcium might govern EPS composition, biofilm architecture, and the underlying regulatory networks in this ecologically significant bacterium are largely unknown. Resolving this is crucial not only for advancing fundamental microbial ecology but also for optimizing the beneficial applications of *P. putida* and mitigating potential detrimental biofilm formation in engineered systems.

We hypothesize that calcium chloride acts as a multifunctional effector in *Pseudomonas putida*, serving dually as a structural modulator of the EPS and as an environmental cue that induces dose-dependent transcriptional changes, leading to altered biofilm development and integrity. To test this, we conducted a comprehensive, multi-scale investigation with three primary objectives: first, to quantify the three-dimensional structural changes and cell distribution in *P. putida* biofilms across a gradient of calcium chloride concentrations using confocal microscopy; second, to examine the biophysical consequences on the EPS matrix, focusing on polysaccharide molecular weight distribution and polydispersity; third, to link these phenotypic changes to the underlying transcriptional responses via genome-wide RNA sequencing. By integrating structural, biophysical, mechanical, and transcriptomic insights, this study seeks to establish a novel mechanistic framework for calcium-mediated biofilm regulation in *P. putida*, thereby challenging the prevailing paradigm and highlighting the critical importance of species-specific adaptation in microbial responses to environmental ions.

## 2 Materials and methods

### 2.1 Bacterial strain, cultivation, and maintenance

The model organism used throughout this study was *Pseudomonas putida* KT2442, a domesticated, plasmid-free derivative of the soil isolate *P. putida* KT2440, renowned for its metabolic versatility and genetic tractability (Wang et al., 2010). Glycerol stocks (25% v/v) of the strain were maintained at −80 °C in Luria-Bertani Broth (LB). For routine cultivation and biofilm experiments, cells were revived by streaking onto LB agar plates supplemented with a maintenance antibiotic cocktail: tetracycline (15 μg/mL), kanamycin (50 μg/mL), and ampicillin (100 μg/mL). All liquid cultures were incubated at 28 °C with continuous shaking at 180 rpm, an optimal temperature for *P. putida* growth (Filipič et al., 2012).

### 2.2 Biofilm cultivation under controlled calcium conditions

A standardized biofilm cultivation protocol was established. A single isolated colony was inoculated in 5 mL of LB medium previously supplemented with antibiotics from a fresh LB agar plate. This pre-culture was grown overnight (~16 h) to late exponential phase (OD_600nm_ ≈ 1.2). For biofilm initiation, this culture was diluted 1:100 into fresh, pre-warmed LB medium containing the same antibiotic concentrations to create a homogeneous working suspension.

To determine the dose-dependence of calcium, 5 mL of the inoculated LB medium was prepared in sterile 50 mL polypropylene centrifuge vials. Calcium chloride dihydrate (CaCl₂·2H₂O, ideal for molecular biology) was added to achieve final concentrations of 0 mM (control), 1.5 mM, 3 mM, and 15 mM. This concentration range was selected to span from ambient (trace) levels to a high ionic strength condition, simulating potential environmental fluctuations and stress. Sterile, pre-cleaned 24 mm diameter circular glass coverslips (Citotest) were aseptically placed at an angle into each tube, ensuring partial submersion to provide a consistent surface for biofilm adhesion. The tubes were gently sealed with sterile cotton plugs to allow for aeration and were incubated at a static temperature of 28 °C for a duration of 48 h. To maintain nutrient availability and remove planktonic cells, the spent medium was carefully aspirated and replaced with 5 mL of fresh, pre-warmed medium (with corresponding CaCl₂ concentrations) at the 24-h mark.

### 2.3 Confocal laser scanning microscopy for architectural analysis

Biofilms were developed for as long as 7 days. Coverslips were collected and processed for confocal microscopy every 24 h. Coverslips were delicately washed by immersing three times in sterile 1X phosphate-buffered saline (PBS, pH 7.4) to eliminate loosely bound cells. Biofilm viability and architecture were assessed using the LIVE/DEAD® BacLight™ Bacterial Viability Kit (Hope et al., 2002). Coverslips were stained in the dark for 15 min with a solution containing 3.34 μM SYTO® 9 (penetrating all cells, fluorescing green) and 20 μM propidium iodide (PI, penetrating only cells with compromised membranes, fluorescing red). Excess stain was removed by a final gentle rinse in PBS.

Biofilm observation was carried out with a Zeiss LSM 980 laser scanning confocal microscope (LSCM), controlled by Airyscan 2 and a 20×/0.8 NA Plan-Apochromat mounted objective. SYTO 9 was excited with a 488 nm laser and emission collected between 500 and 550 nm; PI was excited with a 561 nm laser and emission collected between 600 and 650 nm. For each biological replicate and condition, Z-stacks encompassing the entire biofilm thickness were acquired from at least three random, non-overlapping fields of view. Image stacks were processed and analyzed using a standardized pipeline in BiofilmQ. The BiofilmQ (Hartmann et al., 2021) software packages were utilized for quantitative extraction of key architectural parameters: total biovolume (μm^3^ per μm^2^ of substrate), average and maximum thickness (μm), surface roughness coefficient (a measure of height heterogeneity), and percent substratum coverage. All raw BiofilmQ output files, including complete parameter calculations for each replicate and time point, are provided in the Supplementary material “figure of biofilm parameter data” (XLSX format) and “supp_tables_selected_params” (XLSX format). Data from three independent biological replicates were pooled for statistical analysis.

### 2.4 Extraction and physicochemical characterization of extracellular polymeric substances

The EPS matrix from 48-h biofilms was extracted using a sequential physical–chemical method designed to maximize yield while minimizing cell lysis (Jia et al., 2016). Biofilm biomass from multiple coverslips per condition was carefully scraped into ice-cold 1X PBS and pooled. The cell suspension was mildly sonicated (100 W, total of 10 min pulsed mode: 5 s, on and 5 s off) in an ice bath to break down the biofilm matrix. This was followed by a centrifugation step of the suspension at 12,000 g, 30 min at 4 °C for the recovery of the supernatant (soluble EPS). The pellet was resuspended in fresh prepared PBS, brief vortexing for 30″ followed by a last centrifugation. The combined supernatants constituted the crude EPS extract.

This extract was concentrated and purified via dialysis (3.5 kDa molecular weight cutoff membrane) against deionized water for 48 h at 4 °C, with water changes every 12 h. The dialysate was filter-sterilized through a 0.22 μm pore-size membrane and subsequently lyophilized to obtain dry, crude EPS powder.

### 2.5 Gel permeation chromatography for polysaccharide analysis

To purify the polysaccharide fraction, crude EPS underwent treatment involving the enzymatic degradation of proteins and nucleic acids, where it was dissolved and incubated with DNase I, RNase A, and Proteinase K at 37 °C overnight, followed by heat inactivation and recovery of the polysaccharides through dialysis and lyophilization.

For gel permeation chromatography (GPC) analysis, samples were dissolved at 2 mg/mL in 0.1 M NaNO₃ eluent containing 0.02% sodium azide, filtered (0.45 μm), and injected (50 μL) into an Agilent 1260 Infinity II HPLC system equipped with a Waters Ultrahydrogel linear column (7.8 × 300 mm) and a refractive index (RI) detector. Isocratic elution was conducted utilizing 0.1 M NaNO₃ at a flow rate of 1.0 mL/min and a column temperature of 40 °C, with a calibration curve established via pullulan standards spanning from 5.8 kDa to 708 kDa (Hoagland et al., 2002). Data were processed with Empower™ 3 software to calculate key molecular weight parameters: peak molecular weight (Mp), number-average (Mn), weight-average (Mw), Z-average (Mz), and the polydispersity index (Đ = Mw/Mn).

### 2.6 Atomic force microscopy for nanomechanical mapping

Given the thin-layer characteristics of the 48-h biofilm and the quantitative results from confocal laser scanning microscopy (CLSM), the biofilm for atomic force microscopy (AFM) analysis was extended to 7 days under identical conditions to ensure sufficient biomass for reliable mechanical testing. The biofilm on the coverslip was carefully rinsed with PBS buffer prior to measurement experiments and maintained in a moist state.

Biofilm nanomechanics were probed and quantitatively characterized in the nano-scale with Force Spectroscopy using Bruker Dimension Icon AFM. A ContAl-G probe with a triangular pyramidal tip (nominal half-angle 35°, spring constant 0.12 N/m, calibrated via thermal tune) was used. Force-distance curves were acquired at a minimum of 50 random locations per biofilm sample across three biological replicates. The trigger force applied was configured to 2 nN, with an approach and retract velocity of 1 μm/s.

Raw curves were processed using a custom analysis pipeline in R. The Young’s modulus (stiffness) was extracted by fitting the approach curve to the Hertz contact model for a pyramidal indenter (Baniasadi et al., 2014), assuming a Poisson’s ratio of 0.5 for the hydrated biofilm. The hysteresis ratio, a metric utilized to quantify viscoelastic energy dissipation, was determined by analyzing the area delineated between the loading and unloading curves, which was then normalized against the area of the loading curve. The adhesion force was determined from the minimum force value in the retraction curve. Stringent quality filters (e.g., R^2^ of Hertz fit > 0.3, valid contact point detection) were applied to ensure data reliability.

#### 2.6.1 Rationale for limited sample sizes in biofilm AFM

Bacterial biofilms represent an inherently varied, hydrated viscoelastic matrix. Obtaining artifact-free force curves requires stringent quality control, including unambiguous contact point detection, stable baseline, and high Hertz model fit quality (*R*^2^ > 0.3). The resulting filtered dataset sizes (*n* = 2–10 per condition) reflect this rigorous filtering rather than insufficient measurement effort (a minimum of 50 locations were probed per biological replicate). Similar or smaller sample sizes have been accepted in peer-reviewed biofilm AFM studies when effect sizes are large and trends are corroborated by orthogonal methods (Drame et al., 2021; Pattem et al., 2018; Zeng et al., 2015). Given the ~75% stiffness reduction observed at 3 mM CaCl₂—an effect size far exceeding typical intra-condition variability—and the independent validation by CLSM, GPC, and RNA-seq, we consider these data biologically informative despite the modest *n*.

### 2.7 RNA sequencing and transcriptomic analysis

For transcriptomic profiling, total RNA was isolated from 48-h (Day 2) biofilm samples (cells plus matrix) using the RNeasy PowerBiofilm Kit, which includes a mechanical bead-beating step for efficient biofilm disruption and an on-column DNase I digestion. The integrity of RNA was assessed utilizing an Agilent 2100 Bioanalyzer; exclusively samples exhibiting an RNA Integrity Number (RIN) of 8.0 or greater were employed for the construction of the library. Strand-specific cDNA libraries were prepared and sequenced on an Illumina NovaSeq 6000 platform to generate 150 bp paired-end reads.

The raw sequencing data underwent quality control using FastQC. Adapters and low-quality bases were trimmed with Trimmomatic. High-quality reads were aligned to the *P. putida* KT2440 reference genome (assembly NC_002947.4) employing HISAT2, followed by gene-level read counts generation via featureCounts18; differential expression analysis was conducted using the DESeq2 package in R19, with genes exhibiting an adjusted *p*-value (padj) < 0.05 and an absolute log2 fold change > 1 deemed significantly differentially expressed. We performed Gene Ontology (GO) terms and KEGG pathway functional enhancement analysis for the identified genes using the clusterProfiler package (Yu et al., 2012) to identify biological processes and pathways significantly affected by calcium treatment.

### 2.8 Statistical analysis

All experiments involved a minimum of three independent biological replicates, with CLSM and GPC data expressed as mean ± standard deviation (SD) and analyzed via one-way ANOVA followed by Tukey’s HSD *post-hoc* test using GraphPad Prism (v9.0.0); in contrast, AFM data, reflecting variable high-quality force curves per condition, are reported as mean ± standard error of the mean (SEM). Given the non-normal distribution and unequal variances common in nanomechanical data, group comparisons were assessed using non-parametric Kruskal-Wallis tests, supplemented by Welch’s ANOVA and permutation testing (10,000 iterations) to confirm trends. A *p*-value < 0.05 was considered statistically significant for all analyses unless otherwise specified for transcriptomics (*p*adj < 0.05).

The Shapiro–Wilk test assessed normality for ANOVA, Levene’s test evaluated homogeneity of variance, and the Kruskal-Wallis nonparametric test was employed when assumptions were violated, with the Benjamini-Hochberg adjustment applied to RNA-seq *p*-values.

## 3 Results

**Figure 1:**
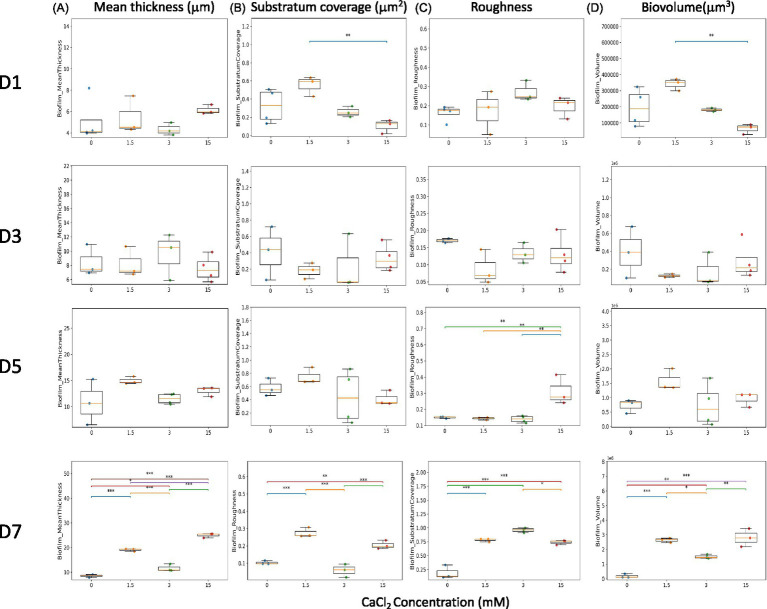
Quantitative analysis of biofilm architectural parameters. Graphical representation of quantitative data extracted from CLSM image stacks using biofilm analysis software. **(A)** Biofilm biovolume (μm^3^/μm^2^) for total, live (SYTO-9) components. **(B)** Biofilm roughness coefficient. **(C)** Substratum coverage (%). **(D)** Average biofilm thickness (μm). Data presented as mean ± SD (*n* = 3); asterisks denote statistical significance (*p* < 0.05) compared to the 0 mM control.

To analyze the stage-specific regulatory role of Ca^2+^ in *P. putida* biofilm development, we employed confocal laser scanning microscopy (CLSM) imaging technology combined with BiofilmQ software to quantitatively characterize biofilms from four CaCl₂ treatment groups (0, 1.5, 3, 15 mM) over days 1 to 7. Time-series results support dividing biofilm development into early (days 1–2), morphogenesis (days 3–5), and maturation (days 6–7) phases: Ca^2+^-related structural differences showed initial differentiation trends in the early phase, amplified during morphogenesis, and consolidated into stable phenotypes during maturation (e.g., highly correlated roughness/specific surface area and substrate coverage metrics exhibited significant time-dependent changes; Figure 1). Based on this staging scheme, we performed RNA sequencing and extracellular polymeric substance molecular weight distribution (GPC-RI) analysis at 48 h (d2) to capture Ca^2+^-induced “initiation” signals in early molecular responses. Atomic force microscopy characterization of mature biofilms at d7 revealed final-state mechanical and adhesion properties. Integrated multiscale data demonstrate that calcium ions initiate molecular reprogramming during early development, ultimately shaping mature mechanical phenotypes through structural remodeling during the plastic phase, establishing a comprehensive developmental framework.

### 3.1 Calcium chloride induces dose-dependent alterations in biofilm architecture

**Figure 2:**
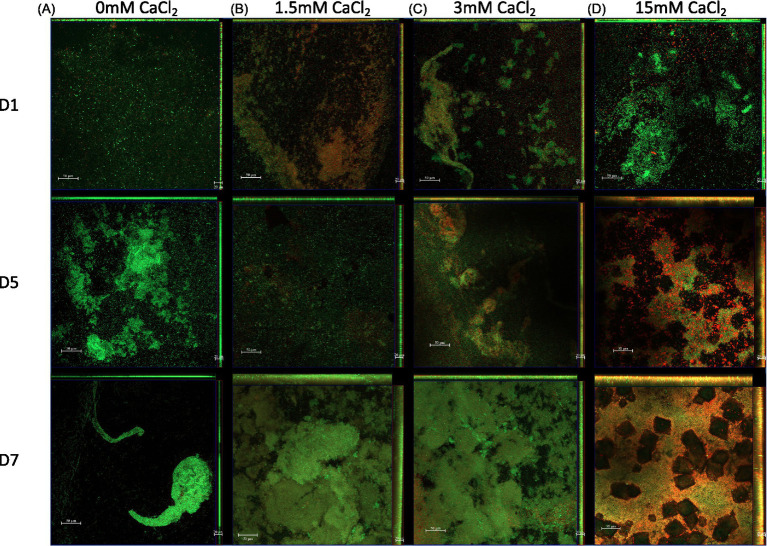
Representative confocal laser scanning microscopy (CLSM) images of D1–D7 *P. putida* biofilms stained with SYTO-9 (green, intact cells) and propidium iodide (red, compromised cells/eDNA). Images show the structural transition from a dispersed morphology at lower concentrations to a thicker, more massive structure at 15 mM CaCl_2_. Scale bar = 20 μm. **(A)** 0 mM, **(B)** 1.5 mM, **(C)** 3 mM, **(D)** 15 mM.

To quantitatively assess the structural impact of calcium ions on *Pseudomonas* biofilm, we performed confocal laser scanning microscopy (CLSM) observations on biofilms cultured for 1–7 days under physiologically relevant CaCl₂ concentration gradients (0, 1.5, 3, and 15 mM). Live-cell staining with SYTO 9 (intact cells, green) and propidium iodide (damaged cells and extracellular DNA, red) enabled simultaneous visualization of cell distribution and matrix-associated nucleic acids. Qualitative analysis revealed concentration-dependent morphological transitions: at the mature stage, the biofilm exhibited significantly increased thickness and a more three-dimensional structure under 15 mM CaCl₂ conditions; while 1.5 mM and 3 mM conditions exhibited typical clustered structures with relatively dispersed architecture, and the biofilm under 1.5 mM CaCl₂ was notably thicker than that at 3 mM (Figure 2).

Quantitative image analysis using BiofilmQ software revealed a significant time-dependent response of live-cell biofilm volume to Ca^2+^ (Figure 1). At d1, significant differences existed among Ca^2+^-treated groups (p_ANOVA < 0.01), with volumes at 3 mM and 15 mM significantly lower than at 0 mM (*p* < 0.01), suggesting early high Ca^2+^ inhibits biofilm formation. Conversely, at d3 and d5, no significant differences were observed between groups (p_ANOVA > 0.05), indicating reduced sensitivity of overall volume to Ca^2+^ at this stage. By d7, significant differences reappeared between groups (p_ANOVA < 0.001). Volumes were significantly enhanced at 1.5–15 mM, peaking at 15 mM, while 3 mM showed markedly limited expansion compared to 1.5 mM (*p* < 0.01). Overall, Ca^2+^ exhibits non-monotonic regulation of biofilm volume: early inhibition, promotion during maturation, and limited expansion at intermediate concentrations (3 mM). This pattern may suggest altered EPS cross-linking or matrix reorganization; however, direct mechanisms were not tested.

The response of live-cell biofilm basal coverage to Ca^2+^ exhibited significant time dependence (Figure 1), similar to Biovolume. At d1, differences between Ca^2+^-treated groups were significant (p_ANOVA < 0.01), with volumes at 3 mM and 15 mM significantly lower than at 0 mM (*p* < 0.01), suggesting an early inhibitory effect of high Ca^2+^ on biofilm formation. Conversely, at d3 and d5, no significant differences were observed between groups (p_ANOVA > 0.05), indicating diminished Ca^2+^ sensitivity in basal coverage at this stage. By d7, significant differences reappeared between groups (p_ANOVA < 0.001). Volumes increased significantly at 1.5–15 mM, peaking at 3 mM, while 1.5 mM and 15 mM were nearly equivalent (*p* < 0.01). This pattern is consistent with the hypothesis that calcium ions influence EPS properties, though alternative explanations involving colloidal stability cannot be excluded (see Discussion).

The response of live biofilm roughness to Ca^2+^ exhibited significant time dependence (Figure 1). At d1 and d3, no significant differences were observed between groups (p_ANOVA > 0.05), indicating low sensitivity of biofilm roughness to Ca^2+^ at this stage. At d3 and d5, significant differences were found between Ca^2+^-treated groups (p_ANOVA < 0.01). At d5, the roughness at 15 mM was significantly greater than other concentrations, which were comparable. At d7, roughness significantly increased at 1.5 mM and 15 mM, while roughness at 3 mM and in the control group showed little change. Overall, calcium ions exerted minimal influence on biofilm roughness during early cultivation. By the maturation phase, calcium ions demonstrated temporal differences in their effects: high concentrations increased roughness immediately, low concentrations produced a delayed effect, while medium concentrations showed little change. This indicates calcium ions affect biofilm roughness in a concentration-dependent and time-differential manner.

Average biofilm thickness showed no sensitivity to calcium ion concentration between d1 and d5 (p_ANOVA > 0.05), but significant differences emerged at d7 (p_ANOVA < 0.001) (Figure 1D). At d7, concentrations ranging from 1.5–15 mM significantly increased average thickness, with 15 mM reaching a peak. In contrast, 3 mM exhibited markedly restricted effects compared to 1.5 and 15 mM (*p* < 0.01). This suggests that mature Ca^2+^ levels favor attachment stability and biomass accumulation, while moderate concentrations disrupt EPS integrity. This pattern confirms that calcium ions influence biofilm development in a concentration-dependent and time-differential manner. Comprehensive statistical summaries and all quantified biofilm parameters for each replicate, time point, and calcium concentration are provided in the Supplementary material “biofilm_stats” (XLSX format).

### 3.2 Calcium reduces the molecular weight and increases polydispersity of matrix polysaccharides

**Figure 3:**
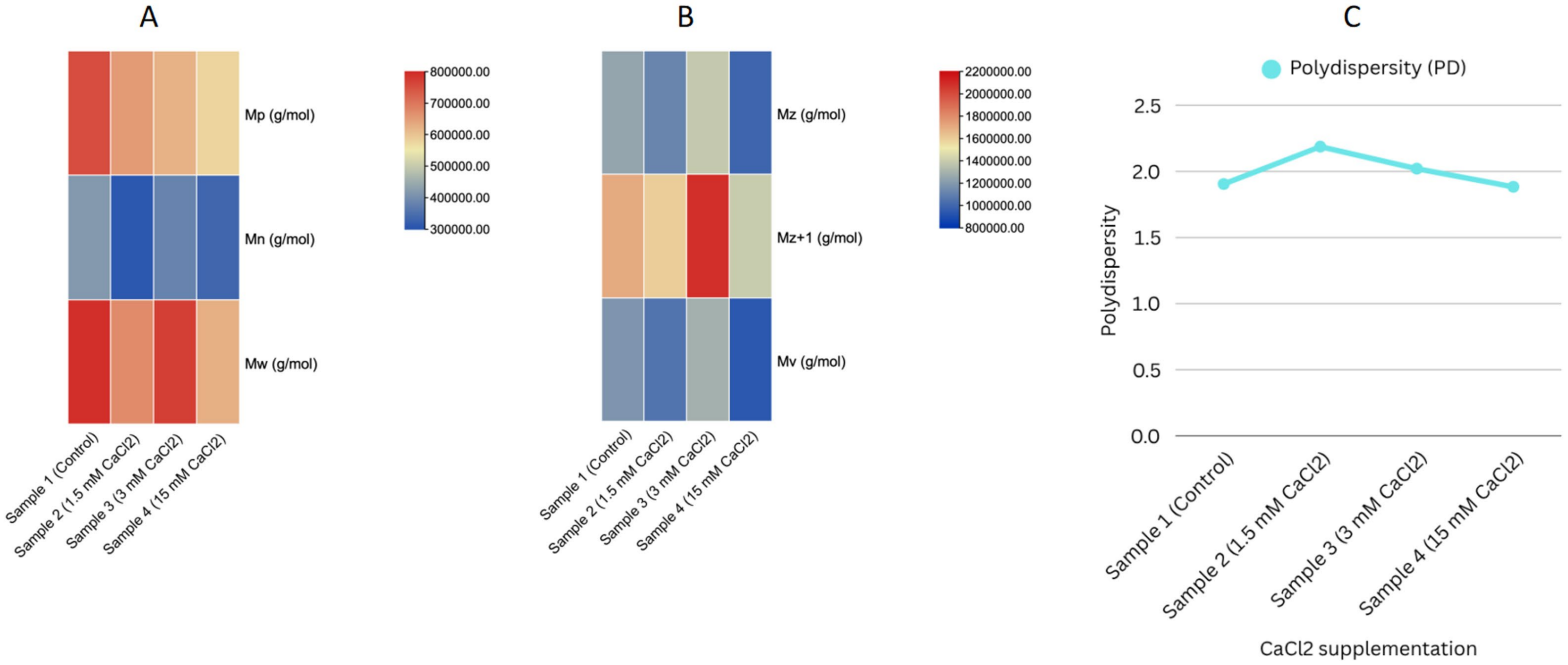
Graphical summary of GPC data. **(A)** Heat map of peak molecular weight (*M*p), number-average molecular weight (*M*n), and weight-average molecular weight (*M*w). **(B)** Heat map of z-average molecular weight (*M*z) and viscosity-average molecular weight (*M*v). **(C)** Line chart showing the trend in polydispersity index (*Đ* = *M*w/*M*n) across CaCl_2_ concentrations. The data demonstrate a general trend of decreasing molecular weight and complex changes in polymer dispersity.

To elucidate the biophysical basis of the architectural changes, we extracted the 48 h biofilm’s extracellular polymeric substances (EPS) and performed high-resolution gel permeation chromatography (GPC) on the purified polysaccharide fraction. This analysis revealed profound, concentration-dependent remodeling of the EPS polymer ensemble (Table 1, Figure 3).

**Table 1:** Molecular weight distribution of EPS polysaccharides.

Parameter	0 mM CaCl_2_	1.5 mM CaCl_2_	3 mM CaCl_2_	15 mM CaCl_2_
Mp (g/mol)	755,194	653,532	624,151	580,624
Mn (g/mol)	417,626	308,704	382,800	333,641
Mw (g/mol)	795,355	674,982	773,421	628,199
Mz (g/mol)	1,247,048	1,117,952	1,387,386	994,895
Mz + 1 (g/mol)	1,710,962	1,603,096	2,092,200	1,400,168
Mv (g/mol)	1,181,081	1,051,864	1,290,421	939,323
Polydispersity (PD)	1.904467	2.186502	2.020431	1.882859

Summary table of molecular weight parameters and polydispersity index (Đ) for polysaccharides extracted from biofilms grown under different CaCl₂ concentrations, as determined by Gel Permeation Chromatography (GPC). Mp (Peak Molecular Weight), Mn (Number-Average Molecular Weight), Mw (Weight-Average Molecular Weight), Mz (Z-Average Molecular Weight), Mv (Viscosity-Average Molecular Weight).

The peak molecular weight, often called Mp, along with the weight-average molecular weight, denoted as Mw, of the matrix polysaccharides showed a steady and continuous decrease in their values as the concentration of CaCl₂ was gradually increased. Mp declined from 755,194 g/mol in the control to 580,624 g/mol at 15 mM, while Mw dropped from 795,355 g/mol to 628,199 g/mol (Figures 3A,B). This consistent reduction suggests that calcium ions either inhibit the synthesis of high-molecular-weight polysaccharides or promote their fragmentation, potentially through chelation of polymer chains or activation of extracellular hydrolases.

In contrast, the Z-average molecular weight (Mz), which is heavily weighted toward the largest polymer species in the distribution, exhibited a non-linear response. It increased from 1,247,048 g/mol at 0 mM to a maximum of 1,387,386 g/mol at 3 mM, before dropping sharply to 994,895 g/mol at 15 mM (Figure 3B). This transient peak in Mz at 3 mM indicates that moderate calcium levels may initially promote the formation of large, supramolecular aggregates or complexes, which are subsequently destabilized under higher ionic strength.

The polydispersity index (Đ = Mw/Mn), reflecting the breadth of the molecular weight distribution, was highest at 1.5 mM (Đ = 2.19), signifying a highly heterogeneous polymer population at this intermediate concentration (Figure 3C). The lower and more similar Đ values at 0 mM (1.90) and 15 mM (1.88) suggest that both the native state and high-calcium stress favor a more uniform, though fundamentally altered, polymer size distribution. Collectively, these data demonstrate that calcium accessibility does not simply cross-link the EPS; it actively remodels the polysaccharide landscape, affecting polymer size, aggregation state, and matrix homogeneity in a complex, dose-dependent fashion.

### 3.3 Calcium chloride compromises biofilm nanomechanical properties

**Figure 4:**
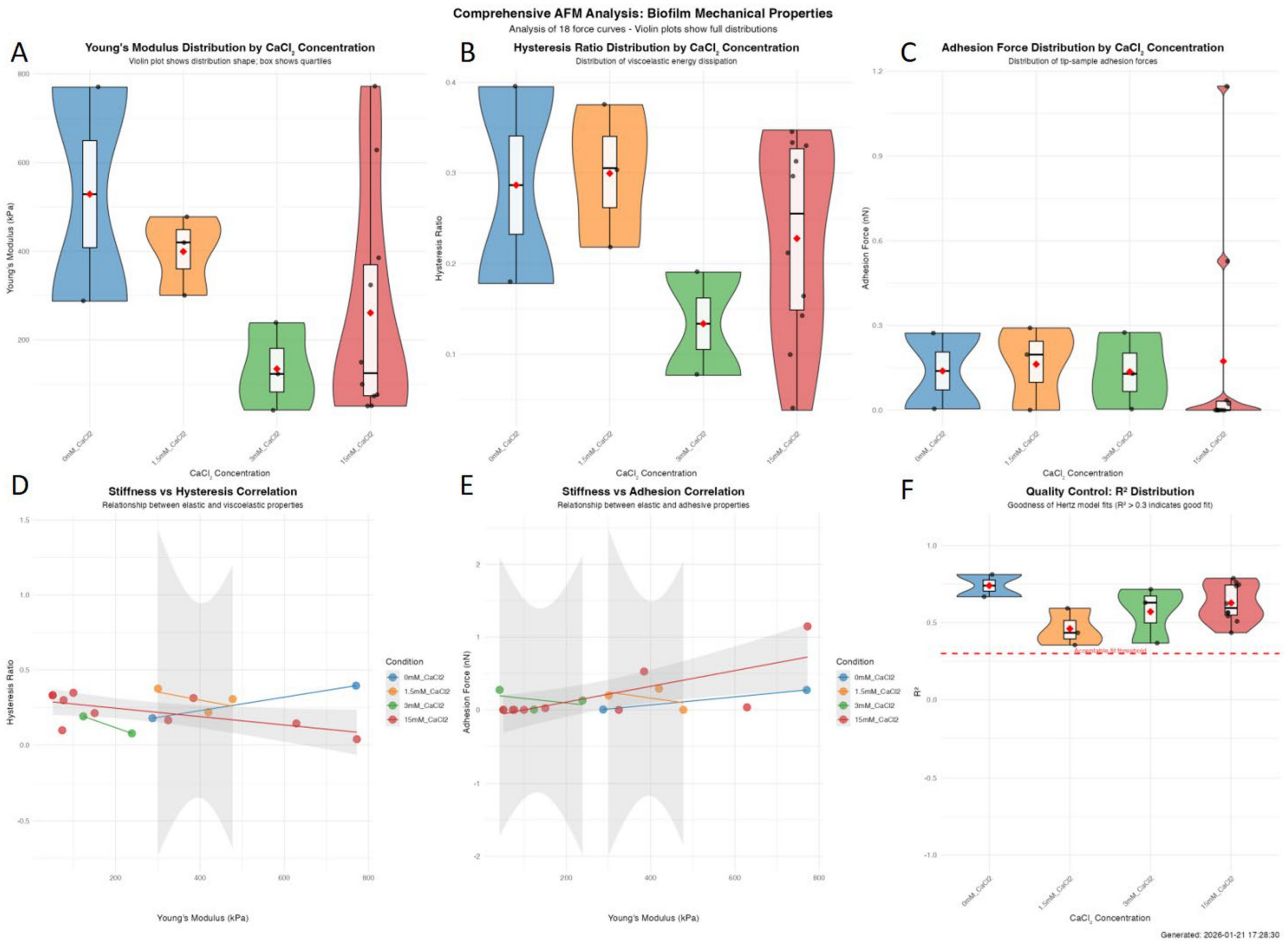
Exploratory nanomechanical characterization of *P. putida* biofilms under calcium stress. **(A)** Young’s modulus across CaCl_2_ concentrations shows a biphasic pattern with maximal softening observed at 3 mM in exploratory measurements. **(B)** Hysteresis ratio indicating viscoelastic energy dissipation follows a parallel trend. **(C)** Adhesion force between the AFM probe and biofilm interface. **(D)** Relationship between stiffness and hysteresis at lower calcium concentrations. **(E)** The correlation of stiffness (Youngs’s modulus) with adhesion force is shown in panel E. **(F)** R-squared distribution for Hertz model fit quality per condition, with a red dashed reference line at R^2^ = 0.3 (the quality filtering threshold). Data presented as mean ± SEM; points represent individual force curves. Important note: Due to stringent quality filtering required for hydrated biofilm AFM, valid force curve numbers per condition are small (*n* = 2–10; see Supplementary Table S1). These data are presented as exploratory and hypothesis-generating. The large effect sizes (~75% stiffness reduction at 3 mM) and independent corroboration by orthogonal methods (CLSM architecture, GPC polymer analysis, RNA-seq) support the biological relevance of the observed directional trends, but quantitative conclusions await validation with larger sample sizes (e.g., high-throughput AFM or rheometry).

Biofilm mechanical characterization by AFM is challenging due to surface heterogeneity and the difficulty of obtaining artifact-free force curves on hydrated, soft materials. After stringent quality filtering (*R*^2^ of Hertz fit > 0.3, valid contact point detection; see Supplementary Table S1), valid force curves were obtained for *n* = 2 (0 mM), *n* = 3 (1.5 mM), *n* = 3 (3 mM), and *n* = 10 (15 mM) curves. Given these limited sample sizes, we present these mechanical data as exploratory and hypothesis-generating. The observed directional trends, particularly the ~75% reduction in median stiffness at 3 mM relative to controls, are presented as supportive evidence that aligns with the more statistically powered architectural, polymer, and transcriptomic datasets, rather than as definitive quantitative measurements. The median Young’s modulus from these exploratory measurements suggested a biphasic pattern, with the lowest values observed at 3 mM CaCl₂ (134.4 ± 57.1 kPa) compared to controls (528.9 ± 241.5 kPa). A partial recovery to 261.0 ± 82.5 kPa was observed at 15 mM. However, given the limited sample sizes (*n* = 2–3 for control and 3 mM conditions), these values should be interpreted with caution and are presented as exploratory observations rather than quantitative conclusions. Moderate calcium levels significantly reduce the biofilm’s load-bearing ability, while the hysteresis ratio, reflecting the energy loss during indentation, displayed a similar non-monotonic pattern. It increased slightly at 1.5 mM (0.300 ± 0.045) from the control (0.287 ± 0.109), then dropped sharply to a minimum at 3 mM (0.134 ± 0.046), before partially recovering at 15 mM (0.228 ± 0.035) (Figure 4B). The marked reduction at 3 mM signals a transition from a dissipative, cohesive hydrogel to a more brittle, less cohesive mechanical regime.

An alternative physical explanation for the increased biofilm thickness at 15 mM CaCl₂ should be considered. High concentrations of divalent cations can reduce the electrical double layer of Gram-negative bacteria, leading to uncontrolled cell aggregation (flocculation) rather than organized biofilm development. Zeta potential measurements would help distinguish between these possibilities (see Limitations).

**Figure 5:**
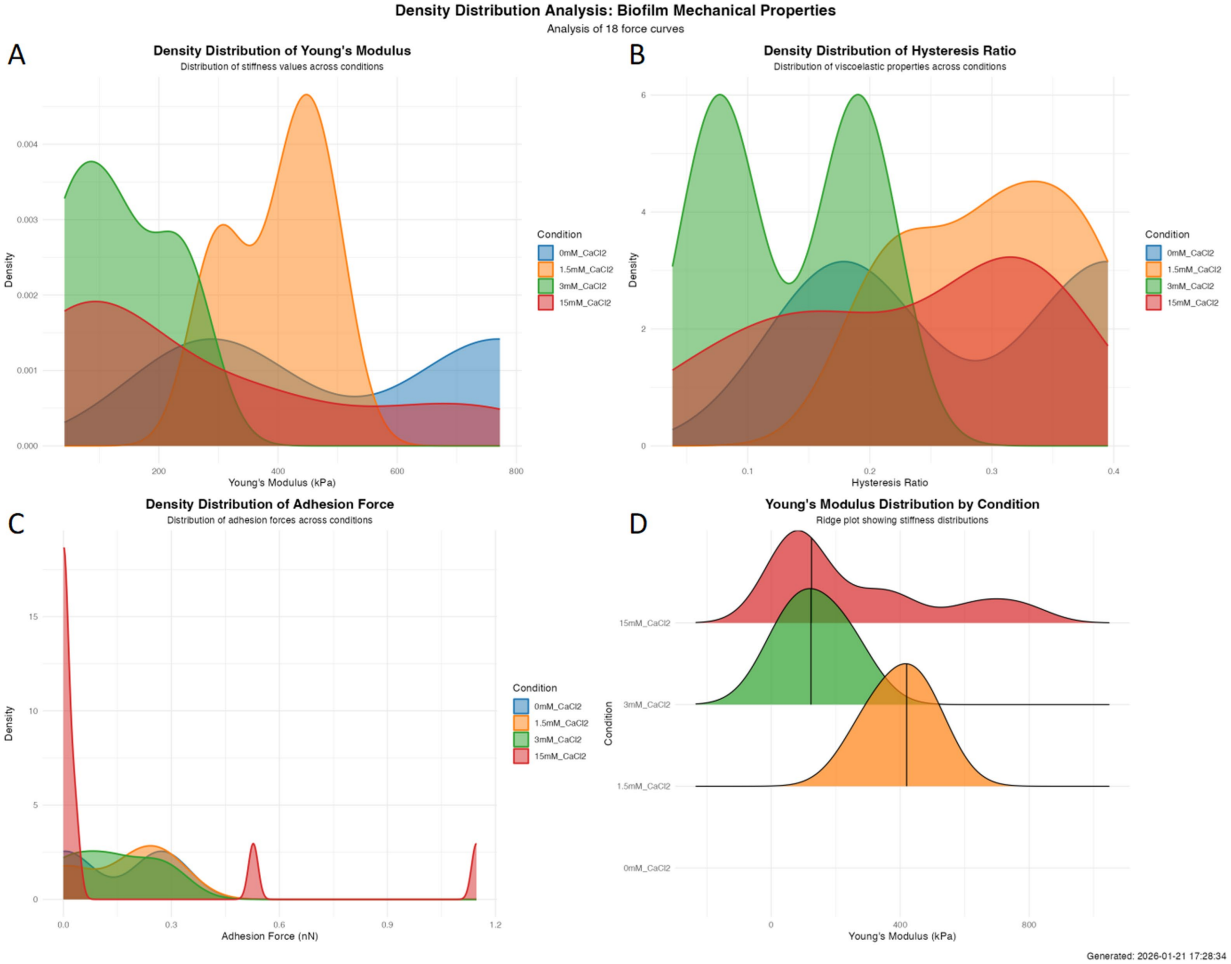
Density distribution analysis of biofilm mechanical properties from AFM force spectroscopy. **(A–C)** Kernel density estimation plots showing the distribution of **(A)** Young’s modulus (stiffness), **(B)** hysteresis ratio, and **(C)** adhesion force for biofilms grown under indicated CaCl_2_ concentrations (0, 1.5, 3, and 15 mM). **(D)** Ridge plot comparing stiffness distributions across conditions. Density plots are shown for visualization of the filtered dataset only and are not intended for statistical inference. Sample sizes (number of valid force curves per condition after quality filtering) are: 0 mM (*n* = 2), 1.5 mM (*n* = 3), 3 mM (*n* = 3), and 15 mM (*n* = 10). See Supplementary Table S1 for individual curve data and filtering criteria. Kernel density estimation requires larger sample sizes than are available here, particularly for the 0 mM, 1.5 mM, and 3 mM conditions. These plots are therefore illustrative of the distribution of the filtered data and should not be overinterpreted. Statistical comparisons of mechanical parameters are reported in Figure 4 and the main text, with the understanding that small sample sizes limit definitive conclusions for Young’s modulus. The exact values for each force curve passing filters are provided in AFM_Filtered_Results.csv.

Adhesion forces between the AFM tip and biofilm surface showed moderate variation, with slight increases at 1.5 mM and 15 mM (~0.2 nN) compared to the control and 3 mM conditions (~0.1 nN) (Figure 4C). Density distribution analysis revealed a progressive narrowing in the variability of Young’s modulus with increasing calcium concentration (Figure 5D), suggesting calcium homogenizes the mechanical landscape, albeit at a greatly reduced stiffness. Correlation analysis suggested an inverse relationship between stiffness and hysteresis at lower calcium levels, consistent with the breakdown of a cohesive viscoelastic network (Figure 4D). While statistical power was limited, the directional consistency across replicates and the alignment with structural and polymer data strongly support the biological significance of these mechanical alterations.

### 3.4 Calcium triggers dose-dependent transcriptional reprogramming

**Figure 6:**
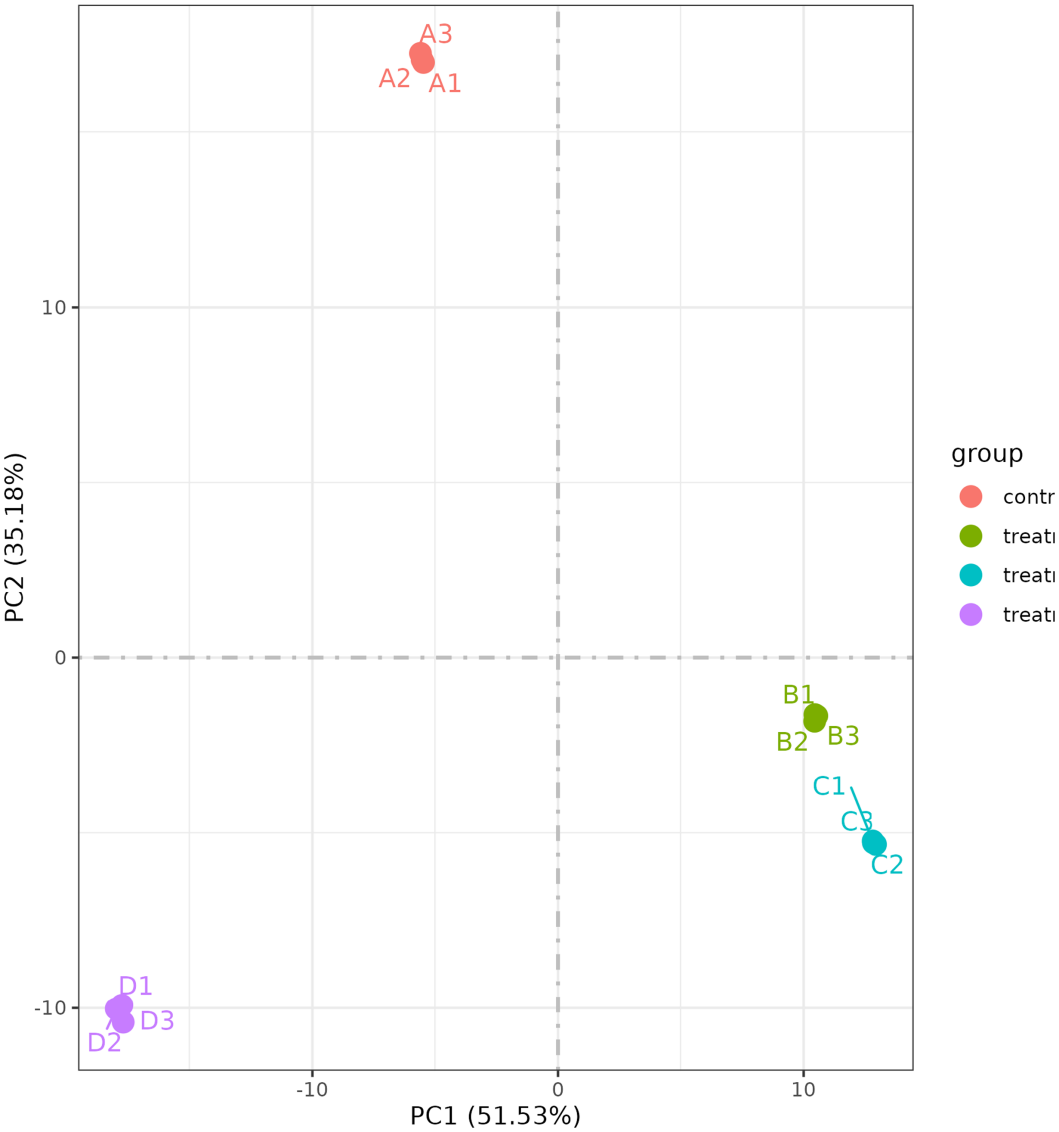
Calcium chloride induces dose-dependent transcriptional reprogramming in *P. putida* biofilms. Principal component analysis (PCA) of RNA-seq data from *P. putida* biofilms grown for 48 h in the presence of 0 mM, 1.5 mM, 3 mM, and 15 mM CaCl_2_. Each point represents a biological replicate (*n* = 3 per group), with ellipses denoting 95% confidence intervals. Clear separation along PC1 (51.53% variance) and PC2 (35.18% variance) indicates that calcium concentration is the primary driver of global gene expression changes.

To uncover the genetic regulatory logic driving the observed phenotypic changes, we performed genome-wide RNA sequencing (RNA-seq) on biofilm samples from all CaCl₂ conditions after 48 h of growth. The full differential expression results for all pairwise comparisons can be found in Supplementary Table S2. Principal Component Analysis (PCA) of the transcriptomic data revealed clear, dose-dependent segregation of the treatment groups, with the first two principal components explaining 86.71% of the total variance (PC1: 51.53%, PC2: 35.18%) (Figure 6). The tight clustering of biological replicates within each condition and their separation along PC1 confirm that calcium concentration is the primary determinant of global gene expression rewiring.

**Figure 7:**
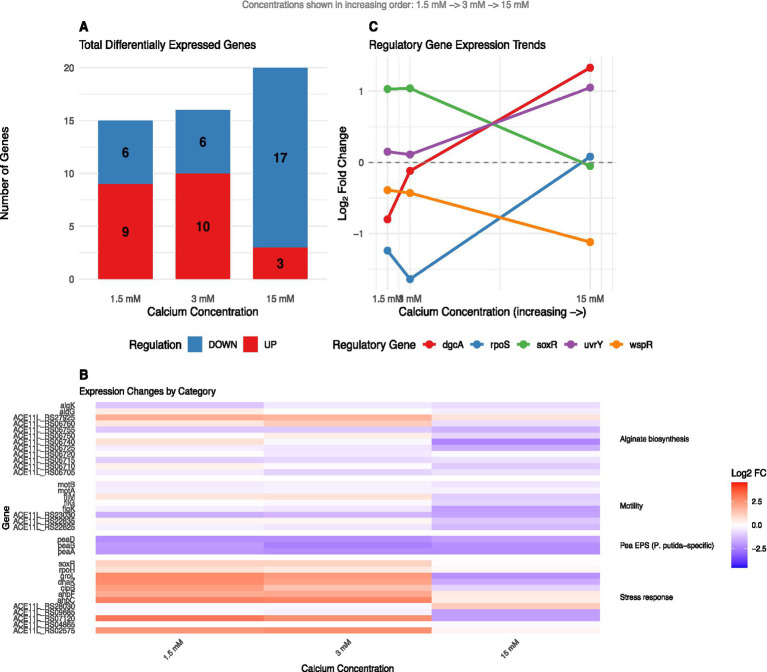
Dose-dependent transcriptional response to calcium in *P. putida* biofilms. **(A)** Number of differentially expressed genes (|log₂FC| > 1, adjusted *p* < 0.05) at each calcium concentration, showing upregulation (red) and downregulation (blue). The response intensifies from 1.5 mM to 3 mM and remains extensive at 15 mM. **(B)** Heatmap showing expression changes (log₂ fold-change) for genes grouped by functional category, including alginate biosynthesis, motility, exopolysaccharide biosynthesis, adhesins, stress response, and ribosomal biogenesis. Calcium concentrations increase from left to right (1.5 mM → 3 mM → 15 mM). **(C)** Expression trends for key regulatory genes (*wspR*, *uvrY*, *soxR*, *rpoS*) across calcium concentrations, demonstrating distinct dose-dependent regulatory patterns.

Differential expression analysis (adjusted *p* < 0.05, |log₂FC| > 1) quantified the scale of this reprogramming. Treatment with 1.5 mM CaCl₂ altered the expression of approximately 3,400 genes (approximately 1,500 upregulated, 1,900 downregulated). This increased to approximately 3,600 genes at 3 mM and remained at approximately 3,600 genes at 15 mM (Figure 7A). A core set of genes showed significant expression changes across all three calcium concentrations (1.5 mM, 3 mM, and 15 mM), defining a conserved calcium-responsive transcriptional network in *P. putida*. The commonality of differentially expressed genes across various conditions is delineated in Supplementary Figure S2.

The transcriptional response showed a marked dose-dependence in intensity and directionality (Figure 7B). At 1.5 mM calcium, the response was relatively moderate, with enrichment of genes involved in cell adhesion and initial stress sensing. At 3 mM calcium, there was a clear reduction in pathways involved in biofilm formation and maintenance, including exopolysaccharide biosynthesis, motility mechanisms, and adhesin production for cell-surface adhesion. At 15 mM calcium, the response shifted toward stress adaptation, with strong upregulation of heat shock proteins, oxidative stress response genes, and ribosomal biogenesis factors.

Examining key regulatory genes revealed distinct dose-dependent expression patterns (Figure 7C). The diguanylate cyclase gene *wspR* showed a biphasic response, while the oxidative stress regulator *soxR* and the stationary phase sigma factor *rpoS* were progressively upregulated with increasing calcium concentration, indicating a shift toward stress adaptation at elevated calcium levels.

**Figure 8:**
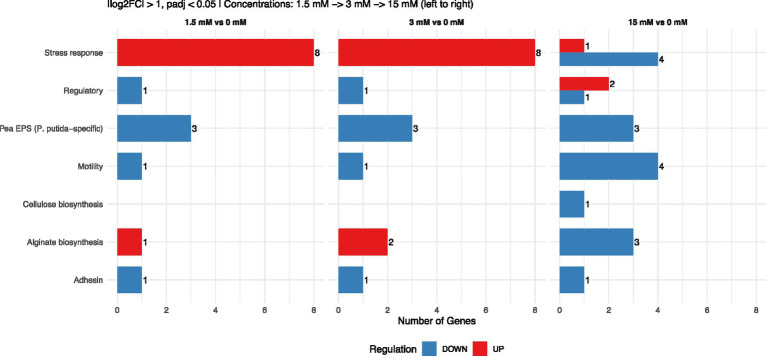
Differentially expressed genes by functional category. Barplot showing the number of upregulated (red) and downregulated (blue) genes in each functional category at 1.5 mM, 3 mM, and 15 mM calcium (categories shown left to right within each concentration). Categories include cell adhesion, motility/chemotaxis, c-di-GMP signaling, biofilm formation, EPS/matrix biosynthesis, regulatory functions, stress response, and transport/metabolism. Data represent DEGs meeting thresholds of |log₂FC| > 1 and adjusted *p* < 0.05.

Categorizing differentially expressed genes functionally demonstrated a consistent, dose-responsive approach (Figure 8). There was a systematic downregulation of pathways and operons critical for biofilm formation and maintenance, including exopolysaccharide biosynthesis, major biofilm adhesins (*lapA*, *lapF*), and flagellar motility genes (*flgK*, *fliG*, *fliM*, *motA*, *motB*). Concurrently, pathways governing stress response (including oxidative stress regulators *ahpC*, *ahpF*, *soxR* and heat shock proteins *dnaK*, *groL*, *clpB*), ribosomal biogenesis, and amino acid biosynthesis were coordinately upregulated. This transcriptional shift depicts a clear physiological trade-off: under calcium stress, *P. putida* downregulates the energetically costly machinery for sessile, multicellular life and upregulates programs for core cellular maintenance, anabolism, and stress resilience, effectively prioritizing survival over community investment.

A different perspective on these transcriptional modifications warrants attention. The upregulation of ribosomal biogenesis and metabolic pathways at 15 mM CaCl₂ could signal increased growth capacity rather than a stress response. Distinguishing between these possibilities requires growth curve measurements under each calcium condition (see Limitations).

### 3.5 Integration of multi-scale findings

Integrating our multi-scale observations across molecular, architectural, mechanical, and transcriptional levels, a coherent picture emerges of calcium’s dose-dependent effects on *P. putida* biofilms. At 1.5 mM calcium, biofilm expansion is promoted with a 35% increase in biovolume, accompanied by a moderate transcriptional response of approximately 3,400 differentially expressed genes. At 3 mM calcium, a critical transition occurs where biofilm architecture collapses with reduced biovolume, thickness, and surface coverage, and exploratory AFM measurements suggest the softest mechanical phenotype, with a ~ 75% reduction in median stiffness observed in the filtered dataset (note: sample sizes were small; see Section 3.3). Approximately 3,600 genes are differentially expressed, particularly with decreased EPS biosynthesis, motility, and adhesin genes. At 15 mM calcium, biofilm thickness increases but biovolume does not fully recover, polysaccharide molecular weight decreases by approximately 20%, and mechanical stiffness only partially recovers. The transcriptional program at 15 mM shifts toward stress adaptation, with upregulation of heat shock proteins, oxidative stress responses, and ribosomal biogenesis, while matrix production genes remain suppressed. This integrated model is consistent with the hypothesis that calcium acts as a dose-dependent environmental signal that triggers species-specific adaptive responses in *P. putida*, fundamentally different from the stabilizing role observed in *P. aeruginosa*.

## 4 Discussion

Our multi-scale time-series analysis reveals that Ca^2+^ exerts a phased influence on *P. putida* biofilms, progressing from early molecular reprogramming to final mechanical alteration. This ‘priming–structural remodeling–final-state fixation’ trajectory suggests that early Ca^2+^ signaling establishes a developmental path that constrains later phenotypic outcomes. This framework moves beyond a static snapshot and provides a dynamic model for how an environmental signal can orchestrate biofilm development over time.

### 4.1 Time-dependent model of *Pseudomonas putida* formation under calcium ion stress

This study demonstrates that Ca^2+^ exerts a distinctly phased effect on *P. putida* biofilms: early-stage (d1–d2) transcriptional responses at the molecular level and rearrangement of EPS molecular weight distribution suggest Ca^2+^ may initiate regulatory processes related to substrate organization, ion homeostasis, and stress adaptation; subsequently, during the morphogenesis phase (d3–d5), quantitative CLSM time-series analysis indicates progressively amplified intergroup differences in structural parameters; Finally, during the maturation phase (d6–d7), these structural trajectory differences became fixed, manifesting as final-state differences in mechanical properties and adhesion (AFM) at d7. Collectively, these multi-scale results support a “priming–structural remodeling–final-state fixation” working model, suggesting that Ca^2+^ influences on the matrix and regulatory networks during early development may shape subsequent mature structural and mechanical phenotypes through path-dependent mechanisms.

### 4.2 Calcium acts as a complex environmental cue, not a universal stabilizer

This multi-scale investigation suggests that the role of calcium in *Pseudomonas putida* biofilm biology differs from the conventional model established for other pseudomonads such as *P. aeruginosa*. Contrary to its well-characterized function as a structural stabilizer in pathogens like *P. aeruginosa*, where Ca^2+^ cross-links alginate to reinforce biofilms, our data reveal that calcium acts in *P. putida* as a multifunctional environmental effector. It dually modulates the physical properties of the extracellular matrix and serves as a potent transcriptional signal, triggering a dose-dependent adaptive response that involves approximately 3,600 genes at elevated concentrations. These findings challenge the generalization of calcium as a universal biofilm cross-linker and suggest that the functional interpretation of ionic signals may depend on species-specific ecological and evolutionary context. The progressive downregulation of biofilm-associated pathways and concurrent upregulation of stress response genes from 3 mM to 15 mM calcium demonstrates that *P. putida* interprets increasing calcium availability as a stress signal rather than an opportunity for structural reinforcement.

The question of whether the observed effects are unique to calcium or indicate a broader reaction to divalent cations is a significant issue that is overlooked by the present experimental framework. Both Ca^2+^ and Mg^2+^ can compress the electrical double layer around bacterial cells, but they may engage different signaling pathways. Without control experiments using equimolar concentrations of MgCl₂, we cannot conclude that calcium acts through specific sensing mechanisms. Consequently, our findings should be interpreted as demonstrating a dose-dependent response to calcium chloride, with the understanding that the specificity of calcium as a signal remains to be established through comparative studies with other divalent cations.

### 4.3 Biphasic structural and mechanical responses reflect matrix remodeling and destabilization

The observed biphasic structure and mechanical response are not mere artifacts but reflect a calcium-ion-driven sequential remodeling process. During the early biofilm formation stage (d1–d2), at concentrations of 3–15 mM, the initial volume reduction and enhanced surface uniformity likely stem from ion-induced densification of the anionic extracellular polymer network, leading to a structurally simplified, high-density biofilm. However, this densification proves mechanically unstable. The concomitant 75% decrease in Young’s modulus and hysteresis observed at 3 mM indicate that this compact structure lacks cohesive strength and viscoelastic integrity. AFM mechanical characterization was limited by low yields of high-quality force curves, particularly for the 0 mM (*n* = 2) and 3 mM (*n* = 3) conditions. Consequently, the reported Young’s modulus values are preliminary and should be interpreted with caution. The mechanical trends are best supported by the more robust hysteresis data and the complementary architectural and polymer analyses. Subsequent volume reduction and thickness increase at 15 mM, coupled with only partial mechanical recovery and high PI signals, indicate lost structural integrity. We interpret this as marking the transition from a functional cell-embedded hydrogel to a brittle composite, rich in crosslinked cell fragments and polymer fragments, exhibiting reduced volume yet nearly complete loss of mechanical elasticity.

### 4.4 Polymer-scale changes underpin mechanical failure and matrix heterogeneity

The monotonic decrease in Mp and Mw directly correlates with loss of stiffness, emphasizing that mechanical robustness in biofilms is intrinsically linked to high-molecular-weight polymer assemblies (Wang et al., 2020; Wloka et al., 2006). This reduction in polysaccharide size likely results from the transcriptional suppression of EPS biosynthetic machinery revealed by RNA-seq, though a potential contribution from calcium-activated extracellular hydrolases cannot be ruled out. The transient peak in Mz at 3 mM is particularly revealing: it suggests calcium initially promotes large, perhaps charge-mediated aggregates. However, the concurrent mechanical minima indicate these aggregates are unstable or improperly integrated, failing to provide cohesive strength. The peak in polydispersity at 1.5 mM reflects a transient state of extreme polymer heterogeneity, which aligns with the observed slight increases in adhesion and hysteresis at this concentration, signs of a disorganized, inconsistently adhesive matrix. The decline in polysaccharide molecular weight could stem from various factors, including the downregulation of EPS biosynthesis genes as indicated by our transcriptome data, with potential involvement of post-transcriptional mechanisms. One possibility is that elevated calcium induces hyper-vesiculation, a phenomenon observed in some Gram-negative bacteria under ionic stress. Outer membrane vesicles (OMVs) can carry hydrolases and exhibit surfactant-like properties that might destabilize the matrix. However, we did not measure OMV production in this study, and this potential mechanism remains speculative. Future investigations quantifying OMV release across the calcium gradient (e.g., via nanoparticle tracking analysis or lipid assays) could establish whether vesiculation contributes to the observed matrix breakdown. Calcium not only disrupts polymers in the EPS but also significantly alters its supramolecular structure, resulting in a weak, brittle, heterogeneous matrix.

### 4.5 Transcriptional reprogramming drives a strategic phenotypic adaptation

The transcriptomic data provide correlative genetic evidence that may explain the observed phenotypic cascade. The coordinated downregulation of EPS-related genes, *lapA*, *lapF*, and flagellar motility genes correlates with the observed reductions in EPS molecular weight, adhesiveness, and mechanical strength. It remains uncertain whether the changes in transcription are the immediate catalysts of phenotypic variation or merely responses to calcium-mediated stress. Simultaneously, the upregulation of ribosomal biogenesis, anabolic pathways, and stress responses (including *ahpC*, *ahpF*, *soxR*, *dnaK*, *groL*, *clpB*, and *rpoS*) suggests a potential metabolic re-prioritization. One interpretation is that *P. putida* perceives elevated calcium levels as a stressor; an alternative interpretation, that divalent cations stimulate growth and cell division, leading indirectly to reduced EPS investment through energetic trade-offs, cannot be excluded based on transcriptomic data alone (see also discussion of growth curve experiments needed in Limitations). The optimal response, based on the transcriptomic data, appears to be a reduction in investment in the biofilm matrix machinery. However, the simultaneous downregulation of flagellar genes (Figure 8) argues against an active dispersal to a planktonic state. Instead, the cells may adopt a metabolically focused, surface-associated but non-matrix-producing state. Whether this represents a distinct physiological state or a transitional phase toward eventual dispersal remains to be determined through functional motility assays and single-cell tracking experiments. This may represent an adaptive strategy tuned to its soil environment, where rapid adaptation to fluctuating conditions is more advantageous than rigid attachment. While our correlative multi-omics data are consistent with the interpretation that the downregulation of these key structural genes is associated with the observed phenotypic remodeling, direct causal relationships remain to be established. Future studies employing targeted genetic knockouts of these operons will be essential to test their necessity for the calcium-induced phenotypic remodeling.

The experiments in this study used a domesticated laboratory strain (*P. putida* KT2442) cultured in rich Luria-Bertani medium. Our findings indicate potential ecological principles that may apply in different scenarios, but caution is essential when relating these conclusions to natural soil ecosystems or the rhizosphere, where numerous environmental and biological factors interact. Future studies using environmental isolates and defined minimal media mimicking soil conditions will be necessary to determine the ecological relevance of these calcium responses.

### 4.6 Ecological implications of species-specific adaptation

The stark contrast between the calcium-induced biofilm destabilization in *P. putida* and the well-documented structural reinforcement in *P. aeruginosa* underscores a fundamental principle of species-specific adaptation. *P. aeruginosa*, adapted to host-associated niches, exploits calcium to fortify persistent, chronic biofilms (Hartmann et al., 2021; Moskowitz et al., 2004). Conversely, *P. putida*, a root-colonizer in dynamic soils (Matilla et al., 2007; Roca et al., 2012), interprets elevated ionic strength as a potential stress signal that triggers a transcriptional and physiological trade-off: diverting resources away from stable biofilm construction and toward cellular maintenance and stress resilience, a strategy likely optimized for survival in fluctuating environments. Its response, to weaken biofilm cohesion and upregulate stress and maintenance pathways, likely enhances fitness by promoting dispersal to more favorable locations (Kaplan, 2010; McDougald et al., 2011) and ensuring cellular survival during fluctuating conditions. This highlights that the role of a ubiquitous ion like calcium cannot be generalized; its biological meaning is decoded through the specific sensory-regulatory networks evolved in each species, reflecting their distinct life history strategies and ecological niches. As such, interpreting elevated calcium as a stress signal to limit biofilm formation may provide a fitness advantage for *P. putida*, preventing irreversible attachment to a potentially unfavorable niche and preserving metabolic flexibility. This adaptation that is specific to the species highlights the essential role of ecological context when inferring the mechanisms that govern biofilm regulation.

### 4.7 Conclusions and future perspectives

In summary, our integrated analysis demonstrates that calcium chloride acts as a multifunctional environmental effector in *P. putida*. It triggers a response that depends on the dosage, initially leading to a compact matrix at lower concentrations, and subsequently resulting in significant transcriptional reprogramming and matrix breakdown at elevated levels. This reprogramming suppresses the production of key adhesins and EPS, leading to architecturally and mechanically deficient biofilms, while concurrently activating stress-resilience and cellular-maintenance pathways. This work suggests that calcium may function not only as a structural ion but also as an active transcriptional and physiological regulator in *P. putida*, emphasizing the species-specific logic of microbial responses to environmental cues.

Future studies should build upon this framework by establishing direct causal links through genetic manipulation (e.g., knockout of *lapA*/*lapF* or EPS genes), expanding the ecological context using flow cells and defined minimal media, and incorporating proteomic/metabolomic analyses to capture post-transcriptional regulation. Furthermore, comparative studies across *Pseudomonas* species will be invaluable for deciphering the evolutionary drivers behind these divergent ionic responses. Such work will not only solidify the mechanistic details revealed here but also enhance our ability to predict and manipulate biofilm behavior in natural and engineered ecosystems.

## 5 Limitations and future perspectives

### 5.1 Experimental limitations

This study hints at significant constraints that must be acknowledged to ensure a comprehensive evaluation of the findings. First, our AFM mechanical characterization yielded limited numbers of valid force curves after stringent quality filtering (*n* = 2–10 per condition). While the large effect sizes and corroboration by orthogonal methods support the directional trends, these mechanical data should be considered exploratory and hypothesis-generating rather than definitive quantitative measurements.

Second, our investigation concentrated on a single laboratory strain (*P. putida* KT2442) cultivated in rich LB medium. Extrapolation to environmental isolates or complex soil/rhizosphere settings requires caution, as calcium effects may be modulated by nutrient availability, pH, flow dynamics, and multi-species interactions.

Third, while our RNA-seq data document transcriptional reprogramming, we did not measure c-di-GMP levels directly, perform functional motility or adhesion assays, or include divalent cation controls (e.g., MgCl₂) to establish calcium specificity. The correlative nature of transcriptomic data means that causation remains to be established through targeted genetic experiments.

Fourth, our study did not include growth curve measurements under each calcium condition, which would help distinguish between stress-response and growth-stimulation interpretations of the transcriptomic data. If 15 mM CaCl₂ increases growth rate or carrying capacity, the “stress response” interpretation would require reconsideration.

### 5.2 Future directions

Future studies should: (i) establish causal links through targeted knockouts of *lapA*, *lapF*, and EPS biosynthesis genes; (ii) measure growth curves and c-di-GMP levels across calcium concentrations; (iii) include MgCl₂ and other divalent cation controls to assess calcium specificity; (iv) perform zeta potential measurements to rule out colloidal instability as an explanation for structural changes at high calcium; (v) quantify OMV production to explore potential mechanisms of matrix destabilization; (vi) conduct functional motility assays (swimming/swarming) to determine whether transcriptional downregulation of flagellar genes translates to reduced motility; and (vii) extend findings to flow cell systems, defined minimal media, and multi-species biofilm models to enhance environmental relevance.

## 6 Conclusion

This study integrates architectural, biophysical, mechanical, and transcriptomic analyses to provide a multi-scale description of calcium chloride effects on *Pseudomonas putida* KT2442 biofilm development. Our data suggest that, contrary to the stabilizing role documented for calcium in *P. aeruginosa*, calcium induces dose-dependent and time-dependent changes in *P. putida* biofilms characterized by reduced EPS molecular weight, compromised mechanical properties (in exploratory AFM measurements), and correlated transcriptional downregulation of matrix-associated genes alongside upregulation of stress and metabolic pathways.

Transcriptomic analysis reveals transcriptional changes that correlate with phenotypic disruption. These correlative data suggest testable hypotheses about genetic mechanisms, but causation remains to be established through targeted genetic experiments. The working model proposed here, that *P. putida* may interpret elevated calcium as a signal to reduce matrix investment, requires validation through targeted genetic and biochemical experiments, including motility assays, growth curve measurements, and divalent cation specificity controls.

These correlative findings challenge the generalization of calcium as a universal biofilm stabilizer and underscore the importance of species-specific investigations. The observed contrast between *P. putida* and the established *P. aeruginosa* paradigm highlights that the role of a ubiquitous ion like calcium cannot be generalized; its biological meaning is decoded through species-specific sensory-regulatory networks that reflect distinct life history strategies and ecological niches.

## Importance

Calcium is widely regarded as a structural stabilizer of bacterial biofilms, particularly in pathogens like *P. aeruginosa*. This study reveals a more nuanced role for calcium in the environmentally relevant bacterium *P. putida*. We show that calcium chloride induces dose-dependent architectural and mechanical remodeling, coupled with extensive transcriptional reprogramming that suppresses matrix production, adhesin expression, and motility while activating cellular maintenance and stress response programs. This study suggests that the common view of calcium as a universal biofilm stabilizer may not extend to all pseudomonads, emphasizing the need for a species-specific perspective on ionic signaling in microbial communities, which is crucial for utilizing *P. putida* in bioremediation, agriculture, and biofilm management.

## Data Availability

The RNA-seq data generated in this study can be found at: https://www.ncbi.nlm.nih.gov/, NCBI BioProject number PRJNA1335146.
